# Modification of the Trizol Method for the Extraction of RNA from *Prorocentrum triestinum* ACIZ_LEM2

**DOI:** 10.3390/ijms25179642

**Published:** 2024-09-06

**Authors:** Ronald Huarachi-Olivera, María Teresa Mata, Alonso Ardiles-Candia, Valentina Escobar-Méndez, Carlos Gatica-Cortes, Matías Ahumada, José Orrego, Boris Vidal-Veuthey, Juan P. Cárdenas, Leonel González, Carlos Riquelme

**Affiliations:** 1 Centro de Bioinnovación Antofagasta (CBIA), Facultad de Ciencias del Mar y de Recursos Biológicos, Universidad de Antofagasta, Antofagasta 1270300, Chile; maria.mata@uantof.cl (M.T.M.); carlos.riquelme@uantof.cl (C.R.); 2 Programa de Doctorado en Ciencias Biológicas, Mención en Biología Celular y Molecular, Facultad de Ciencias de la Salud, Universidad de Antofagasta, Antofagasta 1270300, Chile; 3 Department of Biotechnology, Faculty of Marine Sciences and Biological Resources, University of Antofagasta, Antofagasta 1240000, Chile; 4 Centro de Genómica y Bioinformática, Facultad de Ciencias, Universidad Mayor, Santiago 8580745, Chile

**Keywords:** *Prorocentrum*, HABs, Trizol, RNA isolation, Agilent 4150

## Abstract

In samples of harmful algal blooms (HABs), seawater can contain a high abundance of microorganisms and elemental ions. Along with the hardness of the walls of key HAB dinoflagellates such as *Prorocentrum triestinum*, this makes RNA extraction very difficult. These components interfere with RNA isolation, causing its degradation, in addition to the complex seawater properties of HABs that could hinder RNA isolation for effective RNA sequencing and transcriptome profiling. In this study, an RNA isolation technique was established through the modification of the Trizol method by applying the Micropestle System on cell pellets of *P. triestinum* frozen at −20 °C, obtained from 400 mL of culture with a total of 10^7^ cells/mL. The results of the modified Trizol protocol generated quality RNA samples for transcriptomics sequencing, as determined by their measurement in Analyzer Agilent 4150.

## 1. Introduction

During Harmful Algal Blooms (HABs, also known as “red tides”), dinoflagellates have a significant ecological and environmental impact on marine ecosystems. The ability of dinoflagellates to proliferate rapidly, facilitated by their greater resistance to stressors and possession of a robust cell wall, contributes to the initiation and persistence of HABs. Furthermore, the production of toxins by certain species of dinoflagellates during HABs represents a serious threat to marine life, including fish and shellfish, as well as to human health through the accumulation of toxins in mass-consumed foods. Therefore, understanding the mechanisms that govern the participation of dinoflagellates in HABs is crucial to planning effective monitoring, management, and mitigation strategies in order to address the ecological and economic ramifications of these harmful phenomena. In this regard, the genomic and transcriptomic study of different dinoflagellates such as *Alexandrium* and *Prorocentrum* has gained importance in recent years [1,2,3].

The generation of quality RNA purification protocols for these types of organisms is essential to properly study them. This is achieved by creating efficient and high-throughput transcriptome profiles [4]. There are a number of factors that influence the ease of RNA isolation, including the size and hardness of the cells and cell density in culture, as well as growth habits and cellular products (e.g., mucus) associated with the culture. The most critical step in RNA isolation is the collection and preparation of cellular material [5], where the first step is to release all the RNA by effectively disrupting the tough cell wall of the dinoflagellate without degrading the RNA in the cell. Using an additional technique to disrupt dinoflagellate cells or tissues for maximum yield and high-quality RNA [4] is a crucial requirement in a variety of analyses, including next-generation sequencing, microarrays, and gene expression studies [6]. It is recommended to use samples of RNA with RNA integrity number (RIN) values greater than 5 or 6 for downstream applications. This RNA isolation and evaluation sequence represents a necessary methodological improvement for functional genomic studies in dinoflagellates [7]. Comprehensive transcriptome analysis represents a valuable resource for understanding the molecular metabolic model of dinoflagellates [8]. Dinoflagellate genomes have a unique architecture that may limit their physiological and biochemical responsiveness to environmental stressors [9].

Previous studies have carried out the purification of RNA from algae and dinoflagellates associated with HABs. Trizol has been used for RNA extraction in the frozen tissue of *Pseudopterogorgia elisabethae* and its symbiont *Symbiodinium* sp., resulting in the highest RNA yield compared to extractions obtained with RNeasy [10]. In another RNA extraction protocol, it was found that sand was necessary to effectively grind *Symbiodinium*; the absence of sand during the homogenization process drastically decreased the overall performance. Polyvinylpolypyrrolidone (PVPP) in extraction buffer is crucial for the removal of polyphenolic compounds that can covalently bind to nucleic acids and interfere with many molecular biology procedures [11]. Therefore, one of the most effective isolation techniques is the phenol-chloroform extraction method, which uses organic solvents to isolate the RNA. Another more effective approach used to extract high-quality RNA from HAB environmental samples was with the TransZol Up Plus RNA kit, with a modified protocol [1]. In another investigation, a modified RNA extraction procedure that combines two existing commercial kits, the Trizol and Qiagen RNeasy kits, was efficiently used to recover high-quality RNA under cell homogenization conditions using glass beads at a speed of 4500 rpm for 6 min, resulting in good recovery and preservation of RNA integrity [12]. Considering the above, in the following study, a protocol of the Trizol method for RNA extraction was modified by applying the Micropestle System on frozen cell pellets of a key organism of algal blooms from the coasts of Antofagasta, Chile, the dinoflagellate *Prorocentrum triestinum*, for transcriptomic sequencing, with the purpose of overcoming the methodological difficulties of extraction due to the presence of a large amount of polysaccharides and polyphenols in microalgae in addition to the hardness of the cell wall of the organism.

## 2. Experimental Design

**Figure 1:**
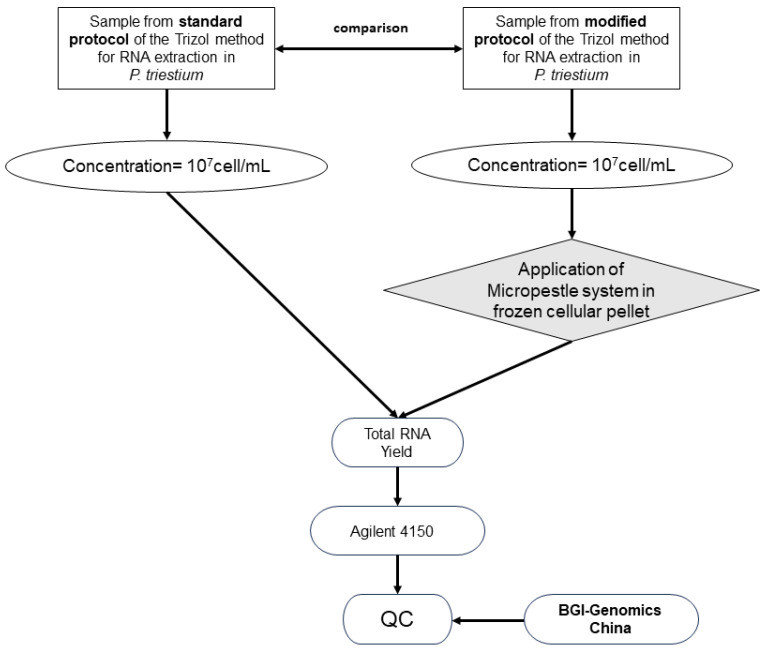
Strategy of the modified protocol of the Trizol method of RNA extraction compared to the standard protocol in *P. triestinum*. QC: Quality Control Evaluation for Transcriptome Sequencing.

The dinoflagellate *Prorocentrum triestinum* strain ACIZ_LEM2 (NCBI accession for 18S rRNA: MN726620) was isolated from the Antofagasta Sea, Chile, (19K 0356231; UTM 7383574). The isolate was obtained from the Centro de Bioinnovación (CBIA) at the University of Antofagasta, Chile. The *P. triestinum* strain was grown in 2 L flat-bottom flasks, supplemented with modified F/2 medium, following the protocol of Guillard and Ryther [13], at 21 °C under 15 µmol photon m^−2^ s^−1^. This research is a prospective experimental study that aims to compare quality control in *P. triestinum* RNA samples from the extraction processes of standard and modified protocols of the Trizol method. It will be based on the evaluations of the values of RNA integrity number (RIN) and 28S/18S ratio established by BGI Genomic China for transcriptomic sequencing processes (Figure 1). The standardized RNA extraction protocol, based on the use of the micropestle system in frozen cell pellets of dinoflagellate HABs such as *P. triestinum* with precise quality controls to perform transcriptomic sequencing, was carried out based on research generated by Zhang and Lin [14]. To achieve this, the cellular biomass of *P. triestinum* was concentrated by centrifugation from 400 mL of culture with a total of 10^7^ cells/mL (information adapted from Schiwitza et al. [15]) because its growth is slow, therefore generating biomass at small volumes. The cell pellets were placed in Trizol and frozen at −80 °C. Then, RNA extraction was carried out, making the modifications in the first steps of the Trizol method with the use of the Micropestle System. In the RNA samples, the mass M (µg), 28S/18S ratio, and RIN were measured using Agilent 4150.

### 2.1. Materials

1. Conical tubes (50 mL) (Greiner Bio-One, Kremsmünster, Upper Austria, Austria, catalog number: 227261).
2. 1.5 mL and 2 mL microtubes (MercK, Darmstadt, Germany).
3. BOECO 2000 mL balloon flask (MercK, Darmstadt, Germany).
4. Analog Timer Timer 24 Hours Adapter (ESHOPANGIE, Santiago, Chile).
5. Micropipette tips (0.5–20 µL, 100–200 µL, 1000 µL) (Gilson™, Middleton, WI, USA).
6. Parafilm (Greenwich, CT, USA 06836)
7. Nitrile Gloves (Winkler, Santiago, Chile).
8. Invitrogen™ TRIzol™ Reagent (Invitrogen, Waltham, CA, USA, Cat. No.: 15596018)
9. Chloroform (Winkler, Lampa, Santiago, Chile, Cat. No.: CL-0595).
10. Ethanol 96% EMSURE^®^ Reag. Ph Eur (MercK, Darmstadt, Germany, Cat. No.: 159010).
11. Iso-PROPYL Alcohol (Winkler, Lampa, Santiago, Chile).
12. Glycogen, RNA grade (Thermo Fisher Scientific, Waltham, CA, USA, Cat. No.: R0561).
13. OmniPur^®^ Water, DEPC Treated, Autoclaved, Nuclease-Free—CAS 7732-18-5—Calbiochem (Merck, Darmstadt, Germany).
14. Face or screen protector.
15. Commercial Bleach, 6% Sodium Hypochlorite (Clorox^®^, Oakland, CA, USA).
16. TAE Buffer, 40X Molecular Biology Grade (Promega, Madison, WI, USA, Cat. No.: V4271).
17. Lafken Agarose (Fermelo Biotec, Santiago, Chile, Cat. No.: FER00AL200G).
18. PAGE GelRed Nucleic Acid Gel Stain 0.1 mL (Biotium, Fremont, CA 94538, USA, Cat. No.: 41008-T).
19. 6X Blue/Orange Dye (Promega, Madison, WI, USA, Cat. No.: G1881).
20. Nuclease-free water (Sigma-Aldrich, Burlington, VT, USA).

### 2.2. Equipment

1. Mini V/PCR vertical laminar flow cabinet (il Telstar, SLU, Barcelona, Spain).
2. KIMBLE^®^ PELLET PESTLE^®^ Cordless Motor (Merck, Darmstadt, Germany).
3. ChemiDoc MP Imaging System (BIO-RAD, Hercules, CA, USA).
  Labnet AccuBlock D1100 Digital Dry Bath (TEquipment.NET, Long Branch, NJ, USA).
  Centrifuges M-240/M-240R (Boeco Germany, Hamburg, Germany).
4. Thermo Sorvall ST 8R centrifuge (Thermo Scientific™, Waltham, MA, USA).
5. Horizontal electrophoresis chamber (Select Bioproducts) (Bio Rad, Hercules, CA, USA).
6. Power source (BioRad, Hercules, CA, USA).
7. BAE224C precision digital analytical balance (Revel) (Velab, Puebla, Mexico).
8. BOECO Vacuum Pump R-300. (Boeco Germany, Hamburg, Germany).
9. Operon Ultra-Low Temperature Freezer −86 °C. (Thermo Fisher Scientific, Waltham, MA, USA).
10. Agilent 4150 (Agilent, Santa Clara, CA, USA).

## 3. Procedure

### 3.1. Test Description

During exponential growth, samples of 400 mL of *P. triestinum* culture were collected, then centrifuged at 5375 rpm at 4 °C for 5 min to obtain 10^7^ cells/mL per cell pellet. The following procedures were performed:

Do not wash cells before adding Trizol™ reagent to avoid mRNA degradation.

1. Resuspend in 750 µL of Trizol Reagent (Invitrogen, Carlsbad, CA, USA) for every 250 µL of cell pellets (obtained from 400 mL of culture with a total of 10^7^ cells/mL) and then store at −80 °C.

The cell pellet with Trizol can be stored at −80 °C for months.

2. Thaw the samples at room temperature and centrifuge at 12,000 rpm for 1 min [14].
3. Transfer the supernatant to a new Eppendorf tube,

Freeze the cell pellet at −20 °C for 2 min or more to achieve efficient homogenization in the following procedure.

**Figure 2:**
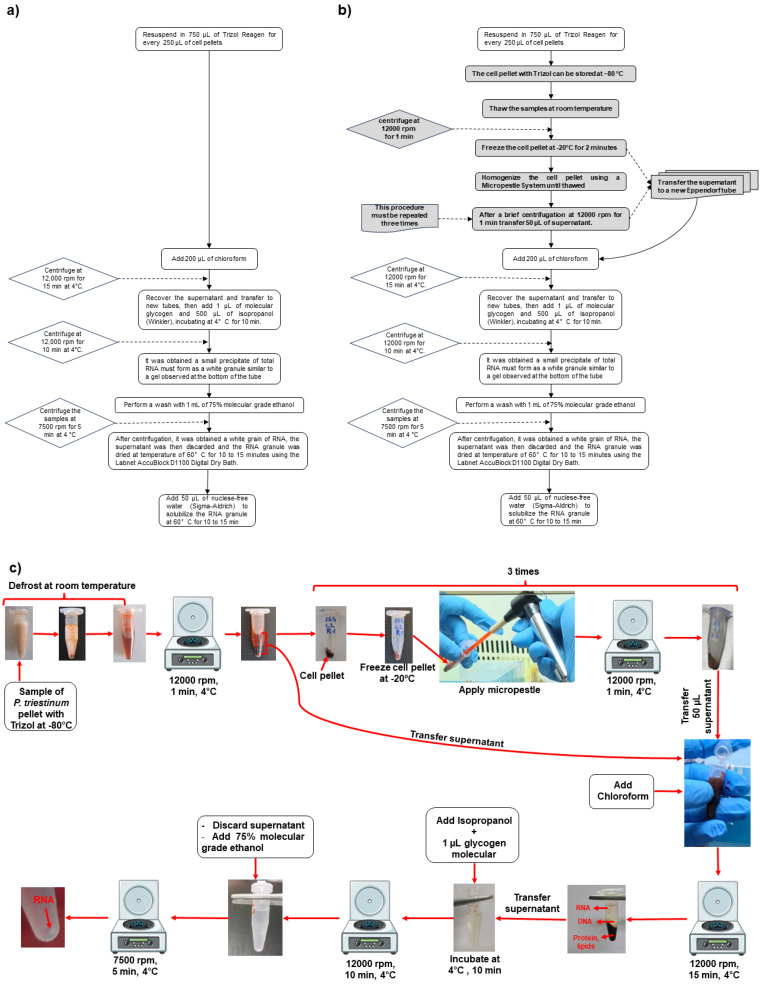
RNA extraction protocol using the Trizol method in *P. triestinum*. (**a**) Flowcharts of the standard RNA extraction protocol and (**b**) the modified RNA extraction protocol where the differences between both protocols are marked in bold and shaded in gray. (**c**) Modified protocol scheme of the Trizol method for RNA extraction in *P. triestinum*. (Information adapted from Zhang and Lin [14]).

4. Homogenize using a Micropestle System (KIMBLE^®^ PELLET PESTLE^®^ Cordless Motor) until thawed [14].
5. After a brief centrifugation at 12,000 rpm for 1 min, transfer 50 µL of supernatant. This procedure must be repeated three times (Figure 2). Then, transfer the cell pellet to the tube with Trizol [14].
6. Add 200 µL of chloroform then centrifuge the samples at 12,000 rpm for 15 min at 4 °C.
7. Recover the supernatant and transfer to new tubes, then add 1 µL of molecular glycogen and 500 µL of isopropanol (Winkler), incubating at 4 °C for 10 min, then centrifuging at 12,000 rpm for 10 min at 4 °C.

After the previous procedure, a small precipitate of total RNA must form as a white granule, similar to a gel, at the bottom of the tube. Next, discard the supernatant very carefully to avoid extracting the white granule.

8. Perform a wash with 1 mL of 75% molecular grade ethanol.

RNA can be stored in 75% ethanol for months at −80 °C.

9. Centrifuge the samples at 7500 rpm for 5 min at 4 °C, observing a white granule of RNA, then discard the supernatant and dry at a temperature of 60 °C for 10 to 15 min using a Labnet AccuBlock D1100 Digital Dry Bath (TEquipment.NET, Long Branch, NJ, USA).

Do not let the RNA pellet dry to ensure complete solubilization of the RNA.

10. Add 50 µL of nuclease-free water (Sigma-Aldrich) to solubilize the RNA granule at 60 °C for 10 to 15 min.

The modifications of the Trizol method are shown in Points 2 to 5 (Figure 2b,c), unlike the standard protocol of the Trizol method (Figure 2a).

### 3.2. Methodologies for Analyzing RNA Quality

**Figure 3:**
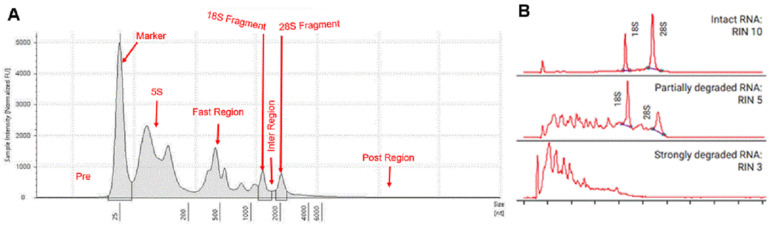
Electropherograms: (**A**) Segments of an electropherogram: The segment preceding the lower marker is called the pre-region. The marker region coincides with the area occupied by the peak of the lower marker. The 5S region covers the small rRNA fragments (5S and 5.8S rRNA, and tRNA). The 18S and 28S regions cover the 18S peak and the 28S peak, respectively. The fast region is located between the 5S region and the 18S region. The inter region is located between region 18S and region 28S. The precursor region covers the precursor RNA that follows the 28S region. Finally, the post region is located beyond the precursor region [17]. (**B**) RIN quality metric with the Bioanalyzer system [20].

Total RNA samples were sent to the laboratory of BGI Tech Solutions (Poland) sp. zoo. where information was generated and processed through electropherograms and the values of method of RNA integrity number (RIN) calculated by Agilent 4150. A total of 50 μL of RNase-free water was added into the sample tubes, which were centrifuged and mixed thoroughly. Samples were taken to evaluate the RIN and the amount of isolated RNA. A two-microliter aliquot of the resulting eluate was analyzed using Agilent 4150. The shape and size of the ribosomal peaks of the 18S and 28S fragments were used for visual assessment of RNA integrity as described in the “manual method” of Strand et al. [16]. The height of the 28S peak provides additional information about the state of the degradation process, that is, during degradation, the 28S band disappears faster than the 18S band. Therefore, it allows the detection of the beginning of degradation [17]. The Agilent 4150 system provides a fast and reliable automated electrophoresis solution for RNA quality control, making it the ideal solution for quality control of biological samples in Next-Generation Sequencing (NGS) [18]. Figure 3A shows the electropherogram reflecting the quality of the RNA according to the following criteria: clear and well-defined peaks of the 18S and 28S fragments of ribosomal RNA, little noise between the peaks, and no or only minimal evidence of low-molecular-weight material [19]. The RIN is calculated using a scale of 1 to 10. A high RIN indicates highly intact RNA and a low RIN suggests a heavily degraded RNA sample [20], as shown in Figure 3B.

**Figure 4:**
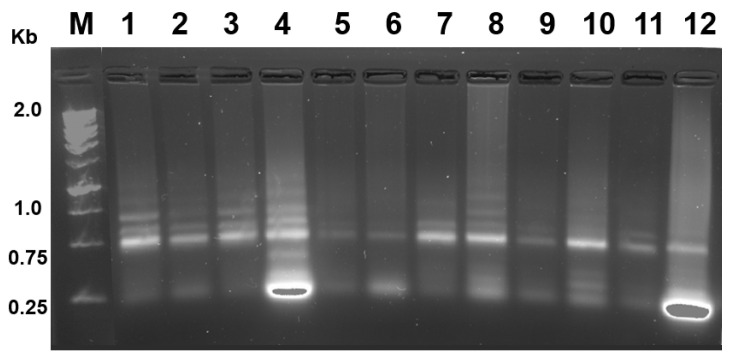
Agarose gel electrophoresis of total RNA extracted from the 12 samples of RNA extracted in *P. triestinum*. The bands denote the major and minor subunits.

A 2% agarose gel was also prepared with 1% Commercial Bleach (Clorox^®^, Oakland, CA, USA) [21] using 1X TAE running buffer and 2 µL of PAGE GelRed Nucleic Acid Gel Stain (Biotium, Fremont, CA, USA). All electrophoretic runs were carried out at 100 V with constant voltage, for a period of 70 min at room temperature. Then, with the use of a ChemiDoc MP Imaging System (Bio-Rad), the characteristic bands of the total RNA were displayed (Figure 4).

## 4. Results

### Yield and Quality of Total RNA Isolated from P. triestinum

Total RNA was isolated using the modified Trizol method protocol, as evidenced by the presence of 28S and 18S bands in 2% agarose electrophoresis gel (Figure 4).

The best results according to Agilent 4150 were samples 5, 6, and 7 (Table 1) with level A according to BGI (meaning that the sample meets the library construction and sequencing requirements). The other results according to the Agilent 4150 system were samples 1, 2, 3, 4, 8, 9, 10, 11, and 12 (Table 1) with level B (the samples do not completely meet the library construction and sequencing requirements but an attempt can be made to construct the library; however, the quality of the sequence is not guaranteed) and samples 13 and 14 with level C (the sample does not meet the library construction and sequencing requirements) being obtained through standard protocols of the Trizol method (Figure 2a).

**Figure 5:**
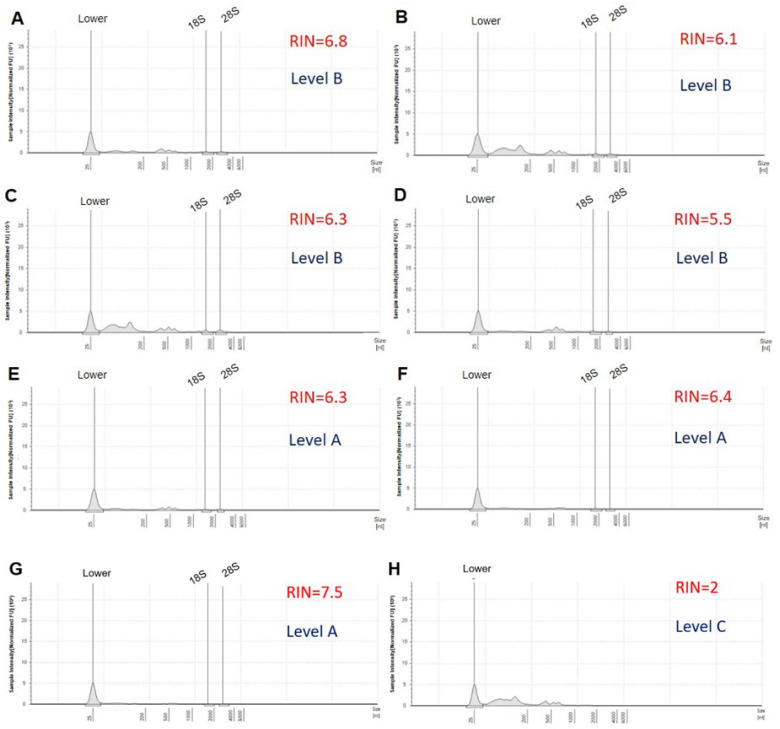
Agilent 4150 Analyzer electropherograms of total RNA extracted from *P. triestinum* samples 1 (**A**), 2 (**B**), 3 (**C**), 4 (**D**), 5 (**E**), 6 (**F**), 7 (**G**), and 13 (**H**) according to Table 1, using the modified Trizol method. Inspection of electropherograms provides additional information to evaluate RNA quality and protocol performance.

**Figure 6:**
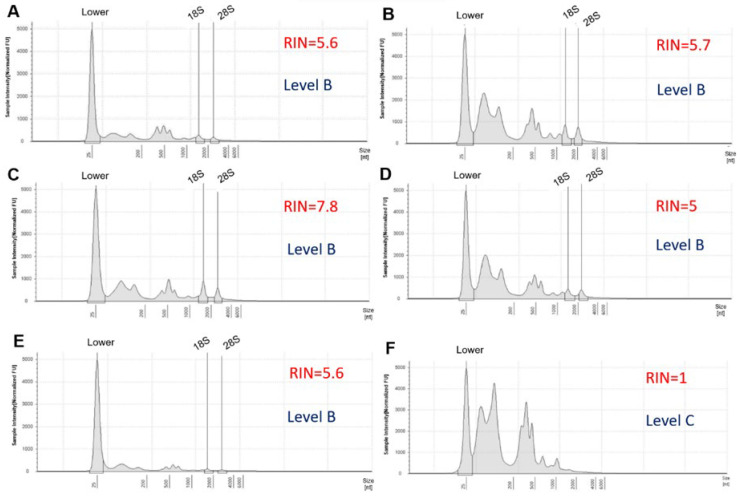
Agilent 4150 Analyzer electropherograms of total RNA extracted from *P. triestinum* samples: 8 (**A**), 9 (**B**), 10 (**C**), 11 (**D**), 12 (**E**), and 14 (**F**) according to Table 1; using the modified Trizol method. The given values of fluorescence intensity are normalized 10^3^.

Figure 5 and Figure 6 show images of total RNA electropherograms processed on an Agilent 4150 system. These were selected from seven and five RNA samples with different RNA integrity (RIN) values. Although the electropherograms of the samples resembled each other in terms of RIN (Figure 5C,E), the differences arose from the observation that the 5S peak was high in sample 3, which had a RIN equal to 6.3 and a level of B, compared to sample 5 with a RIN = 6.3 and a level of A (Table 1). According to the Analyzer electropherograms, the total RNA obtained from samples 5, 6, and 7 showed the highest quality of level A, with RIN > 6; 28S/18S > 0.8 and an absent 5S peak. The expected RIN value (the ratio between the 28S area and the 18S area calculated by the software) for good-quality RNA should be above 9 (or in some cases above 6) [7]. Therefore, the quality of the extracted RNA was efficiently verified using an automated system with Agilent 4150, which demonstrated RINs ranging between 5 and 7.8 (Figure 5 and Figure 6) and RNA quantities of 3.39 µg to 39.9 µg, unlike low-quality samples of level-C RNA with RINs from 0 to 1 and absent 28S and 18S peaks (Table 1, Figure 5H and Figure 6F). The Agilent 4150 Analyzer also validated the integrity of the RNA by observing two peaks showing 28S and 18S rRNA. Table 1 summarizes the quality of RNA extracted from the 12 *P. triestinum* samples using the modified Trizol protocol. The quality control of the RNA samples met the necessary requirements for next-generation transcriptomic sequencing, although in samples with level B, it could generate only bias in the read map. The RNA integrity number (RIN) is calculated using a dedicated software algorithm to evaluate the quality of RNA preparations. The RIN tool is an important step in standardizing user-independent RNA assessments and provides more meaningful information than simple ratio calculations for ribosomal RNA peaks [20].

## 5. Discussion

In this study, the extraction of RNA from the dinoflagellate *P. triestinum* isolated from a coastal bloom that occurred near the city of Antofagasta, located in the Atacama Desert, Chile, was standardized. This particular bloom began in late November 2018 and continued until February 2019. The bloom extended more than 30 km from the coast to the open sea and more than 600 km along the coast from the northernmost city in Chile to the south of Antofagasta, as reported by Ávalos et al. [22]. Therefore, it should be noted that there is difficulty in extracting RNA from *P. triestinum* due to the hardness of the cell wall. This is because dinoflagellates have an outer covering, called the theca or amphiesma, with an outer membrane and a thecal vesicle. Thecal plates contain primarily cellulose and polysaccharides, with a small amount of proteins residing in the cell wall, and an outer membrane of phytoplankton [23]. In environmental HAB samples for RNAseq sequencing, RNA extraction is not straightforward due to the presence of a large amount of polysaccharides and polyphenols in microalgae [1]. In this study, a modified protocol of the Trizol method was established for RNA extraction in *P. triestinum*. To our knowledge, this is the first study in which the modification of the Trizol method is established by applying the Micropestle System in *P. triestinum* cell pellets at −20 °C. The cell pellets were obtained from 400 mL of culture with a total of 10^7^ cells/mL. Meanwhile, research has described the dinoflagellate *Alexandrium catenella*, which is an ideal model species for microalgae that is difficult to lyse due to its resistant cellulose cell wall, and has determined that microgun crushing and sonication are the most reliable methods for the most crucial step in RNA extraction [7]. This research is unlike other research performed on *P. minimum* for RNA isolation, where the Microspestle System was applied to cell pellets frozen with dry ice [14].

*P. triestinum* RNA measurements were evaluated using electropherograms with Analyzer Agilent 4150, with levels A and B being the most reliable for transcriptomic sequencing (Table 1). In general, RIN values greater than 5 were recommended to represent samples with preserved RNA integrity. A typical high-quality RNA electropherogram is shown with a RIN = 7.5, including a clearly visible 28/18S rRNA peak ratio and a small 5S RNA peak, while the partially degraded sample with a RIN = 4.5 was indicated by a shift in the electropherogram to shorter fragment sizes producing a decrease in the fluorescence signal [24]. However, several studies have suggested that researchers should not blindly rely on the RIN number to indicate chloroplast presence, since rRNA from dinoflagellates can interfere with RIN calculations. The generated electropherogram must be interpreted manually to detect the presence of a clear 28S/18S rRNA peak. A flat inter region between those regions represents good RNA quality [4]. The 28S/18S ratio may reflect nonspecific damage to RNA; however, the standard 28S/18S rRNA ratio of 2.0 is difficult to meet, especially for RNA derived from clinical samples [25]. That is why the Agilent 4150 system provides a fast and reliable automated electrophoresis solution for RNA quality control [18]. To obtain reliable data in gene expression analysis, a good method for RNA extraction must be established, followed by constant monitoring of RNA quality during experimentation. High-throughput automated microcapillary electrophoresis (Agilent 2100 Bioanalyzer) can analyze up to 12 RNA samples of dinoflagellate *Symbiodinium* sp. per assay and provide a complete picture of RNA integrity in picograms of RNA quantity compared to conventional methods such as agarose gel electrophoresis or the use of a UV spectrophotometer [12]. For example, in a Electropherogram of total RNA isolated from decidual cells of the placenta and using an automatic gel electrophoresis system in Agilent 4150 with a concentration of RNA in the sample of 1.3 ng/μL, the RIN of 6.7 indicates that the positions of the 18S ribosomal RNA and 28S peaks are of good quality for transcriptomic sequencing [18].

## 6. Conclusions

For transcriptome sequencing in environmental samples, it is essential to standardize a specific protocol of RNA isolation. The objective of this study was to establish a suitable protocol for the isolation of total RNA from samples of *P. triestinum*, a HAB dinoflagellate. The modified Trizol method protocol of applying a Micropestle System on frozen pellets was shown to be effective in providing quality, reproducible RNA that meets standard criteria for transcriptomic sequencing. Extracted RNA with a RIN ≥ 5 in *P. triestinum* is of sufficient quality to produce a library, ensuring a successful RNA sequencing experiment.

## Figures and Tables

**Table 1:** Values of total RNA extracted from *P. triestinum* with Agilent 4150.

Samples	(C) ng/µL	(V) µL	(M) µg	RIN	28S/18S	Level	Remark
1	127	33	4.19	6.8	0.9	B	5S peak is high
2	187	89	16.64	6.1	0.8	B	5S peak is high
3	310	89	27.59	6.3	1	B	5S peak is high
4	203	35	7.11	5.5	0.6	B	RIN < 6; 28S/18S < 0.8
5	195	30	5.85	6.3	1	A	
6	165	31	5.12	6.4	0.8	A	
7	311	84	26.12	7.5	1.1	A	
8	183.07	35	6.41	5.6	0.7	B	RIN < 6; 28S/18S < 0.8
9	331.14	60	19.87	5.7	0.9	B	RIN < 6; 5S peak is high
10	420.00	95	39.90	7.8	0.6	B	28S/18S < 0.8; 5S peak is high
11	296.78	65	19.29	5	0.8	B	RIN < 6; 28S/18S < 0.8; 5S peak is high
12	94.22	33	3.39	5.6	0.7	B	RIN < 6; 28S/18S < 0.8; 5S peak is high
13	221	32	7.07	2	0	C	RIN < 6; 28S/18S < 0.8
14	545.98	32	17.47	1	0	C	RIN < 6; 28S/18S < 0.8; 5S peak is high

C = Concentration, V = Volume, M = Mass. Level according to BGI.

## Data Availability

Data available upon request.
